# Development and external validation of a breast cancer absolute risk prediction model in Chinese population

**DOI:** 10.1186/s13058-021-01439-2

**Published:** 2021-05-29

**Authors:** Yuting Han, Jun Lv, Canqing Yu, Yu Guo, Zheng Bian, Yizhen Hu, Ling Yang, Yiping Chen, Huaidong Du, Fangyuan Zhao, Wanqing Wen, Xiao-Ou Shu, Yongbing Xiang, Yu-Tang Gao, Wei Zheng, Hong Guo, Peng Liang, Junshi Chen, Zhengming Chen, Dezheng Huo, Liming Li, on behalf of the China Kadoorie Biobank Collaborative Group, Junshi Chen, Zhengming Chen, Robert Clarke, Rory Collins, Yu Guo, Liming Li, Jun Lv, Richard Peto, Robin Walters, Daniel Avery, Ruth Boxall, Derrick Bennett, Yumei Chang, Yiping Chen, Zhengming Chen, Robert Clarke, Huaidong Du, Simon Gilbert, Alex Hacker, Mike Hill, Michael Holmes, Andri Iona, Christiana Kartsonaki, Rene Kerosi, Ling Kong, Om Kurmi, Garry Lancaster, Sarah Lewington, Kuang Lin, John McDonnell, Iona Millwood, Qunhua Nie, Jayakrishnan Radhakrishnan, Paul Ryder, Sam Sansome, Dan Schmidt, Paul Sherliker, Rajani Sohoni, Becky Stevens, Iain Turnbull, Robin Walters, Jenny Wang, Lin Wang, Neil Wright, Ling Yang, Xiaoming Yang, Zheng Bian, Yu Guo, Xiao Han, Can Hou, Jun Lv, Pei Pei, Chao Liu, Canqing Yu, Zengchang Pang, Ruqin Gao, Shanpeng Li, Shaojie Wang, Yongmei Liu, Ranran Du, Yajing Zang, Liang Cheng, Xiaocao Tian, Hua Zhang, Yaoming Zhai, Feng Ning, Xiaohui Sun, Feifei Li, Silu Lv, Junzheng Wang, Wei Hou, Mingyuan Zeng, Ge Jiang, Xue Zhou, Liqiu Yang, Hui He, Bo Yu, Yanjie Li, Qinai Xu, Quan Kang, Ziyan Guo, Dan Wang, Ximin Hu, Jinyan Chen, Yan Fu, Zhenwang Fu, Xiaohuan Wang, Min Weng, Zhendong Guo, Shukuan Wu, Yilei Li, Huimei Li, Zhifang Fu, Ming Wu, Yonglin Zhou, Jinyi Zhou, Ran Tao, Jie Yang, Jian Su, Fang Liu, Jun Zhang, Yihe Hu, Yan Lu, Liangcai Ma, Aiyu Tang, Shuo Zhang, Jianrong Jin, Jingchao Liu, Zhenzhu Tang, Naying Chen, Ying Huang, Mingqiang Li, Jinhuai Meng, Rong Pan, Qilian Jiang, Jian Lan, Yun Liu, Liuping Wei, Liyuan Zhou, Ningyu Chen, Ping Wang, Fanwen Meng, Yulu Qin, Sisi Wang, Xianping Wu, Ningmei Zhang, Xiaofang Chen, Weiwei Zhou, Guojin Luo, Jianguo Li, Xiaofang Chen, Xunfu Zhong, Jiaqiu Liu, Qiang Sun, Pengfei Ge, Xiaolan Ren, Caixia Dong, Hui Zhang, Enke Mao, Xiaoping Wang, Tao Wang, Xi Zhang, Ding Zhang, Gang Zhou, Shixian Feng, Liang Chang, Lei Fan, Yulian Gao, Tianyou He, Huarong Sun, Pan He, Chen Hu, Xukui Zhang, Huifang Wu, Pan He, Min Yu, Ruying Hu, Hao Wang, Yijian Qian, Chunmei Wang, Kaixu Xie, Lingli Chen, Yidan Zhang, Dongxia Pan, Qijun Gu, Yuelong Huang, Biyun Chen, Li Yin, Huilin Liu, Zhongxi Fu, Qiaohua Xu, Xin Xu, Hao Zhang, Huajun Long, Xianzhi Li, Libo Zhang, Zhe Qiu

**Affiliations:** 1 Department of Epidemiology and Biostatistics, School of Public Health, Peking University Health Science Center, 38 Xueyuan Road, Beijing, 100191 China; 2 Key Laboratory of Molecular Cardiovascular Sciences (Peking University), Ministry of Education, Beijing, China; 3 Peking University Institute of Environmental Medicine, Beijing, China; 4 Chinese Academy of Medical Sciences, Beijing, China; 5 Medical Research Council Population Health Research Unit at the University of Oxford, Oxford, UK; 6 Clinical Trial Service Unit & Epidemiological Studies Unit (CTSU), Nuffield Department of Population Health, University of Oxford, Oxford, UK; 7 Department of Public Health Sciences, The University of Chicago, 5841 S. Maryland Ave., MC2000, Chicago, IL 60637 USA; 8 Division of Epidemiology, Department of Medicine, Vanderbilt-Ingram Cancer Center, Vanderbilt University Medical Center, Nashville, TN USA; 9 State Key Laboratory of Oncogene and Related Genes & Department of Epidemiology, Shanghai Cancer Institute, Shanghai Jiaotong University School of Medicine, Shanghai, China; 10 Medical department, Liuyang Hospital of Traditional Chinese Medicine, Liuyang, China; 11 People’s Hospital of Liuyang, Liuyang, China; 12 China National Center for Food Safety Risk Assessment, Beijing, China

**Keywords:** Breast cancer, Global health, Prediction model, Absolute risk, Prospective cohort study

## Abstract

**Backgrounds:**

In contrast to developed countries, breast cancer in China is characterized by a rapidly escalating incidence rate in the past two decades, lower survival rate, and vast geographic variation. However, there is no validated risk prediction model in China to aid early detection yet.

**Methods:**

A large nationwide prospective cohort, China Kadoorie Biobank (CKB), was used to evaluate relative and attributable risks of invasive breast cancer. A total of 300,824 women free of any prior cancer were recruited during 2004–2008 and followed up to Dec 31, 2016. Cox models were used to identify breast cancer risk factors and build a relative risk model. Absolute risks were calculated by incorporating national age- and residence-specific breast cancer incidence and non-breast cancer mortality rates. We used an independent large prospective cohort, Shanghai Women’s Health Study (SWHS), with 73,203 women to externally validate the calibration and discriminating accuracy.

**Results:**

During a median of 10.2 years of follow-up in the CKB, 2287 cases were observed. The final model included age, residence area, education, BMI, height, family history of overall cancer, parity, and age at menarche. The model was well-calibrated in both the CKB and the SWHS, yielding expected/observed (*E/O*) ratios of 1.01 (95% confidence interval (CI), 0.94–1.09) and 0.94 (95% CI, 0.89–0.99), respectively. After eliminating the effect of age and residence, the model maintained moderate but comparable discriminating accuracy compared with those of some previous externally validated models. The adjusted areas under the receiver operating curve (AUC) were 0.634 (95% CI, 0.608–0.661) and 0.585 (95% CI, 0.564–0.605) in the CKB and the SWHS, respectively.

**Conclusions:**

Based only on non-laboratory predictors, our model has a good calibration and moderate discriminating capacity. The model may serve as a useful tool to raise individuals’ awareness and aid risk-stratified screening and prevention strategies.

**Supplementary Information:**

The online version contains supplementary material available at 10.1186/s13058-021-01439-2.

## Introduction

Breast cancer is the most common and rapidly increasing female malignancy in China [1]. Compared with developed countries, breast cancer in China is characterized by a rapidly increasing incidence rate, lower survival rate, and vast geographic variation. The annual percent increase in breast cancer incidence was 4.5% and 9.1% in urban and rural areas of China, respectively [2]. In 2015, there were 304,000 newly diagnosed cases and 70,000 deaths from breast cancer, with an incidence rate of 54.3 per 100,000 in urban areas and 34.5 per 100,000 in rural areas [3]. The 5-year relative survival rates during 2003–2015 only ranged from 73.1% to 82.0% in Chinese women (55.9% to 72.9% for rural women), which were much worse than that of 90% for American women [4]. Early detection is the cornerstone of preventing morbidity and mortality due to breast cancer. However, it was impeded by the lack of individuals’ awareness and national scale screening program.

Following the pioneering model derived by Gail et al. in 1989 [5], multiple models have been developed [6]. However, most models were developed in the western populations and may not be applicable to Chinese women, even the Gail model modified for Chinese-Americans [7]. A previous meta-analysis showed that these models tended to overestimate the risk of Asia women [8], and some predictors, such as the number of prior breast biopsies, are not available for most Chinese women. Several models have also been developed in China [9–15]. However, most of them were developed using a case-control design, which is subjected to selection and recall bias. Additionally, all these studies were conducted with participants from the eastern provinces of China, where breast cancer incidence rates are higher than those in the other areas of China [1]. More importantly, of the seven models, only one, which was conducted in Shandong province, has been externally validated in a small cohort with only 34 cases. Therefore, a validated breast cancer risk prediction model based on data from Chinese women with good generalizability is more than timely and much needed.

In this study, we used data from a large nationwide prospective cohort, the China Kadoorie Biobank (CKB), as well as national age- and residence (urban and rural)-specific invasive breast cancer incidence rates and non-breast cancer mortality rates to develop a risk prediction model considering competing risk, and used data from another large prospective cohort, the Shanghai Women’s Health Study (SWHS), to independently validated the model.

## Methods

### Data for model development

Data from the CKB, a large-scale prospective study, was used to derive the relative risk (RR) model [16]. The study took place in 10 study sites, 5 in urban area (Qingdao, Harbin, Haikou, Suzhou, Liuzhou) and 5 in rural area (Pengzhou, Tianshui, Hui county, Tongxiang, Liuyang) of China. The regions were selected according to local disease patterns, exposure to certain risk factors, population stability, quality of death and disease registries, local commitment, and capacity. Potential eligible participants were identified through official residential records. Invitation letters (with study information leaflets) were delivered door-to-door by local community leaders or health workers. The estimated population response rate was ~ 30% (26–38% in the five rural areas and 16~50% in the five urban areas). Overall, a total of 512,715 participants aged 30–79 years old, including 302,510 (59.0%) women were recruited during 2004–2008. All participants had completed a questionnaire and had physical measurements taken.

Incident cases of invasive breast cancer and mortality were identified chiefly through the linkage with the national health insurance claim database and disease registries, supplemented with local residential records and annual active confirmation. The International Classification of Diseases, 10th Revision was used to code all breast cancer (C50) by trained staff who were blinded to baseline information. We excluded women who had missing data for any reproductive factors or who provided implausible data on age at menarche or age at first live birth. We further excluded women who reported previous histories of cancer at baseline or had missing data for body mass index (BMI), leaving 300,824 women in the analysis.

### Data for external validation

Independent data from the SWHS was used to externally validate the derived model based on CKB data [17]. In brief, 74,942 women were recruited from seven urban communities in Shanghai, China during 1996–2000.

At baseline, all information involved in the current analysis was collected through in-person interviews and anthropometric measures following standard protocol. Incident breast cancer cases (ICD-9 code 174) were identified by a combination of active re-surveys every 2 to 4 years and annual linkage with the Shanghai Cancer Registry and the Shanghai death certificate registry. The cancer diagnosis was verified through home visits and reviews of medical charts obtained from the hospitals where the patients were diagnosed. Applying the same exclusion criteria as the CKB data, 73,203 SWHS participants were included.

### Statistical Methods

#### Relative risk prediction model

Participants were considered at risk from the enrollment to the diagnose of invasive breast cancer, death, loss to follow-up, or Dec 31, 2016, whichever came first. Cox proportional hazards model was used to estimate the hazard ratios as the metric of relative risk (RR) for each variable in the model, with age as the timescale, and stratified jointly by 10 study sites and age at enrollment in a 5-year interval (i.e., 100 strata to control the confounding by age and study sites).

We initially considered the following variables to construct the model: education, tobacco smoking, alcohol drinking, total physical activity, consumption of soybean, BMI, height, first-degree family history of overall cancer, menopausal status, number of live birth, age at menarche, total duration of breastfeeding, and usage of contraceptives. Because we did not collect information on family history of breast cancer, we used the family history of overall cancer as a surrogate. The continuous variables were converted to categorical variables to reduce overfitting. Cutoffs of BMI were chosen according to the well-established criteria for Chinese [18]. And, the quartile of height was used as cutoffs of height. For other predictors, cutoffs were chosen when the model achieved the smallest Bayesian Information Criterion (BIC). We assessed the proportional hazards assumption by the Schoenfeld residuals. In line with previous studies [19, 20], we found only BMI was subject to time-varying effects. Therefore, we further split follow-up time into two age intervals at 50 years and added an interaction term of attained age and BMI. We first assessed all variables with *P* < 0.05 together in the model. Variable selection was repeated using stepwise backward elimination, which yielded the same result. The variables were converted to ordinal variables if their RRs were proportional to levels and no evidence of nonlinearity was detected using fractional polynomials. All first-order interactions were tested one by one using the likelihood ratio test comparing models with and without the interaction term. For all variables in the final model, the lowest risk category was regarded as the reference group, to facilitate population attributable risk (PAR) computation.

Given the higher incidence rate of breast cancer in urban areas than that in rural areas, we also tempted to build residence (urban/rural)-specific models, i.e., variable selection and predictors coefficients were separately done in urban and rural datasets. Interestingly, we found that the relative risks were similar between urban and rural areas, and there was no significant interaction between area and risk factors (see Additional file 1). Therefore, we used the same set of relative risk estimates for all participants in the CKB to maintain model parsimony and to more reliably estimate hazard ratios.

#### Absolute risk projection

We used an approach similar to that described by Gail et al. to project absolute risk from initial age to final ag e[5, 21]. Briefly, the absolute risk that a woman who is age *a* and who has risk factors *x* will develop breast cancer by age *a* + *τ* is

1 $$ \mathrm{P}\left(a,\tau, x\right)={\int}_a^{a+\tau }{h}_1\left(t,x\right)\exp \left[-{\int}_a^t\left({h}_1\left(u,x\right)+{h}_2(u)\right) du\right]\mathrm{d}t\kern0.5em $$

where *h*_1_(*t*, *x*) is the age-specific hazards of developing breast cancer and *h*_2_(*t*) is the age-specific hazards for competing causes at age *t*. We can estimate *h*_1_(*t*, *x*) = *h*_10_(*t*)RR(*x*) as the product of age-, residence-specific baseline hazards *h*_10_(*t*) and relative risks RR(*x*) from the relative risk model described above. RR(*x*) are age-constant for all risk factors *x* except for BMI, which has two different RR for < 50 and ≥ 50 years old.

To have a robust and generalizable model, we calculated the baseline age- and residence-specific hazards *h*_10_(*t*), by multiplying age-specific incidence rates in 2014 from the National Central Cancer Registry of China (NCCR) [22] by one minus population attributable risk (PAR). The PAR was estimated using the formula described by Bruzzi et al. [23] and can be interpreted as the fraction in the incidence of breast cancer that would have been reduced during follow-up if all six predictors in the relative risk model (i.e., education, BMI, height, family history of overall cancer, parity, and age at menarche) took the lowest risk category of predictors. PAR of 1 indicates all breast cancer incidence attribute to the factors, while PAR of 0 indicates no breast cancer incidence attribute to these factors. The distribution of risk factors in four groups defined by attained ages (below/above 50 years old) and residence (urban/rural) were different, so we estimated the PAR separately in the four above-mentioned groups. Further, death from causes other than breast cancer will prevent the occurrence of breast cancer, of which risk increased with age. To account for the competing risk, we calculated age- and residence-specific mortality rates of non-breast cancer, *h*_2_(*t*), as age- and residence-specific all-cause mortality rates in 2014 from Health Statistics Yearbook [24] minus age- and residence-specific breast cancer mortality rates in 2014 from the NCCR. These incidence and mortality rates are listed in Additional file 2.

As a sensitivity analysis, we built an absolute risk model using breast cancer incidence rates and non-breast cancer mortality rates from the CKB cohort to understand calibration of internal validation. As another sensitivity analysis, we built an absolute risk model using breast cancer incidence rates and non-breast cancer mortality rates from Shanghai in the external validation (calibrated model) to evaluate whether robust local rates, if available, can improve model performance.

### Validation

The above development process was first done using whole CKB data and repeated in a random two-thirds of the CKB data (derivation subcohort). We found that the same set of predictors was selected and the RRs for predictors were similar using the above-mentioned two methods (Additional file 3). We used data splitting approach for internal validation, i.e., the model was fitted to random two-thirds of the CKB data and evaluated on the remaining one-third (test subcohort). To have more precise estimations of model parameters, we still used the model developed from the whole CKB dataset for external validation in the SWHS dataset. We assessed calibration by comparing the expected number of breast cancer cases (*E*) with the observed number (*O*) overall and for subgroups defined by predictors. The calibration plot was drawn to examine the agreement across deciles of predicted risk in the total population. The projected probability of breast cancer was calculated from the age at enrollment to the younger of either the age at last follow-up or the age on Dec 31, 2016, for the CKB participants or Dec 31, 2014, for the SWHS participants. The 10-year projected risk was also estimated. The 95% confidence intervals (CIs) of *E*/*O* ratios were calculated based on Poisson distribution. An *E*/*O* ratio above one indicates that the model overestimates cancer risk, and an *E*/*O* less than one indicates that the model underestimates cancer risk. Discrimination was quantified by calculating the area under the receiver-operating characteristic curve (AUC), also known as c-statistics, for 10-year risk model. Age- and residence-adjusted AUC was also assessed to eliminate the effect of age and residence. Higher AUC indicates higher discriminating ability, where random classification results in an AUC of 0.5 and perfect discrimination results in 1. To further assess the discriminating accuracy, we estimated the RRs comparing different quintiles of predicted risk. We also estimated a range of performance indices corresponding to a series of cut-offs ranging from 0.4% to 2% in both the CKB and the SWHS. The indices included percent of high-risk population, sensitivity, specificity, positive/negative predictive value (PPV/NPV), and numbers needed to be screened to confirm one case in the next 10 years (NNS, one divided by the PPV).

The calculation of absolute risk was performed using SAS (version 9.4, SAS Institute Inc.), and all other statistical analyses were performed using Stata (version 14, StataCorp).

## Results

Of the 300,824 women in the CKB cohort included in the RR model development, the mean age at recruitment was 51.4 years. Compared with those in rural areas, women in urban areas were older, more educated, more overweight or obese, taller, and were more likely to have positive overall cancer family history, early age at menarche, and less likely to have multiple children (Table 1). Compared with women in urban areas of the CKB, women in the SWHS had similar ages, BMI, and number of live births, but tended to be more educated, taller, to have more relatives diagnosed with cancer, and to have an earlier age at menarche.

**Table 1:** Baseline characteristics of women by residence and dataset in China Kadoorie Biobank (CKB) and Shanghai Women’s Health Study (SWHS)

	CKB				SWHS
	Rural		Urban		Urban
	Derivation	Validation	Derivation	Validation	
No. of participants, n	111,346	55,612	89,204	44,662	73,203
Cases, n	529	267	1007	484	1409
Age in years, mean (SD) and %					
Continuous, years	50.5 (10.2)	50.5 (10.2)	52.6 (10.7)	52.6 (10.7)	52.5 (9.1)
30-	2.4	2.4	1.7	1.5	--
35-	15.8	15.7	11.6	11.4	--
40-	18.3	18.4	15.9	16.1	28.3
45-	13.2	13.2	14.2	14.6	20.8
50-	17.6	17.5	17.1	17.4	14.1
55-	13.6	13.6	13.5	13.4	11.1
60-	8.9	9.0	9.7	9.3	12.8
65-	6.1	5.9	9.1	9.2	13.3
70-	3.7	3.8	6.4	6.4	0.6
75-	0.4	0.4	0.8	0.8	--
Highest education, %					
No formal school	31.9	31.5	17.3	17.1	10.6
Primary school	40.3	40.5	20.4	20.0	10.8
Middle school	21.6	21.6	29.9	30.4	37.2
High school	5.6	5.8	23.2	23.3	28.0
College/university	0.6	0.6	9.2	9.1	13.5
Ever smoker, %	5.6	5.5	4.4	4.6	2.8
Ever weekly drinker, %	3.2	3.2	2.6	2.6	2.3
BMI, mean (SD) and %					
Continuous, kg/m^2^	23.5 (3.4)	23.5 (3.4)	24.2 (3.5)	24.3 (3.5)	24.0 (3.4)
< 18.5	5.2	5.1	3.2	3.0	3.4
18.5–23.9	53.8	53.4	46.6	46.5	49.9
24.0–27.9	31.2	31.6	36.1	36.3	34.6
≥ 28	9.9	9.9	14.0	14.2	12.2
Height, mean (SD) and %					
Continuous, cm	153.2 (5.9)	153.2 (5.9)	155.3 (5.9)	155.3 (5.9)	157.5 (5.5)
< 150.2	29.4	29.4	19.3	19.1	10.3
150.2–154.1	26.3	26.1	23.6	23.5	17.6
154.2–158.1	24.1	24.3	26.1	26.1	27.9
≥ 158.2	20.2	20.1	31.0	31.3	44.2
No. of affected first-degree relatives, %					
0	85.2	85.5	80.6	80.4	74.7
1	13.0	12.7	16.8	16.9	21.5
≥2	1.8	1.9	2.6	2.7	3.8
Postmenopausal, %	50.2	50.2	55.0	55.3	49.2
No. of live birth, mean (SD) and %					
Continuous	2.5 (1.4)	2.5 (1.4)	1.9 (1.2)	1.9 (1.2)	1.8 (1.2)
Nulliparous	0.8	0.9	1.9	1.9	3.3
1	21.1	21.3	50.8	51.2	54.9
2	38.6	38.2	23.6	23.2	21.1
≥ 3	39.4	39.6	23.7	23.7	20.8
Age at menarche, mean (SD) and %					
Continuous, years	15.6 (1.9)	15.5 (1.9)	15.3 (2.0)	15.3 (2.0)	14.9 (1.7)
< 12	4.7	4.7	6.4	6.4	6.3
13–14	26.3	26.4	30.3	30.3	36.5
15–16	38.3	38.4	36.1	36.1	39.5
≥ 17	30.6	30.5	27.2	27.3	17.8
Total months of breastfeeding, mean (SD) and %					
Continuous, months	42.4 (32.2)	42.5 (32.4)	24.0 (22.8)	23.9 (22.5)	15.5 (18.3)
0	2.2	2.4	6.3	6.3	20.2
1–23.9	36.5	36.5	64.1	64.1	56.6
24–35.9	17.4	17.2	12.1	12.1	9.9
36–47.9	14.2	14.0	7.0	7.1	5.9
≥ 48	29.6	29.9	10.4	10.3	7.4
Pill use, %					
Never	91.2	91.4	88.7	88.8	78.6
Ever	8.8	8.6	11.3	11.2	20.4

Abbreviations: *SD* standard deviation, *MET* metabolic equivalent of task, *BMI* body mass index

During a median of 10.2 years of follow-up in the CKB, 2287 women developed invasive breast cancer. The final age- and study site-stratified model included education, BMI, height, family history of cancer, parity, and age at menarche (Table 2). The association between BMI and breast cancer risk was non-significant in women younger than 50 years and was positive associated in women above this age (test-for-interaction was significant). No other significant interaction between predictors was found. Based on the relative risk model and distribution of risk factors, the PARs estimated in urban areas were 0.74 for women younger than 50 years and 0.76 for women 50 years and older. The corresponding PAR estimates in rural areas were 0.63 and 0.65, reflecting fewer cases were attributed to the six predictors in the relative risk model in the rural areas.

**Table 2:** Age- and study site-stratified RR (95% CI) for breast cancer in China Kadoorie Biobank

	Cases	Cases/PYs	RR (95% CI)
		(/100,000)	
Highest education			
No formal school	339	44.64	1.00 (reference)
Primary school	570	60.18	1.17 (1.11 to 1.23)
Middle school	653	84.82	1.37 (1.24 to 1.51)
High school	505	124.5	1.60 (1.39 to 1.85)
College/university	220	165.2	1.87 (1.54 to 2.27)
BMI at age < 50 years, kg/m^2^			
< 18.5	21	98.83	1.00 (reference)
18.5–23.9	357	126.42	0.95 (0.84 to 1.07)
24.0–27.9	187	160.42	0.90 (0.71 to 1.14)
≥28	43	135.81	0.85 (0.60 to 1.21)
BMI at age ≥ 50 years, kg/m^2^			
< 18.5	36	41.07	1.00 (reference)
18.5–23.9	642	66.66	1.25 (1.17 to 1.33)
24.0–27.9	683	94.59	1.57 (1.38 to 1.78)
≥28	318	118.27	1.96 (1.62 to 2.38)
Height, cm			
< 150.2	382	51.58	1.00 (reference)
150.2–154.1	504	66.66	1.13 (1.09 to 1.18)
154.2–158.1	596	78.54	1.28 (1.18 to 1.39)
≥ 158.2	805	105.96	1.45 (1.28 to 1.65)
No. of first-degree relatives diagnosed with overall cancer			
0	1795	71.63	1.00 (reference)
1	402	90.71	1.10 (0.99 to 1.23)
≥ 2	90	136.09	1.57 (1.27 to 1.95)
No. of live birth			
Nulliparous	45	112.79	1.78 (1.29 to 2.45)
1	1067	101.84	1.66 (1.40 to 1.96)
2	719	74.19	1.41 (1.22 to 1.62)
≥ 3	456	47.58	1.00 (reference)
Age at menarche, years			
< 12	187	114.7	1.52 (1.31 to 1.76)
13–14	748	88.12	1.32 (1.20 to 1.46)
15–16	837	74.27	1.15 (1.09 to 1.21)
≥ 17	515	58.77	1.00 (reference)

Abbreviations: *BMI* body mass index, *PY* person-year, *RRs* relative risk, *CI* confidence interval

Cox model was stratified by age at enrollment in 5-year interval (10 groups) and 10 study sites. All predictors above were included in the final model

**Fig. 1:**
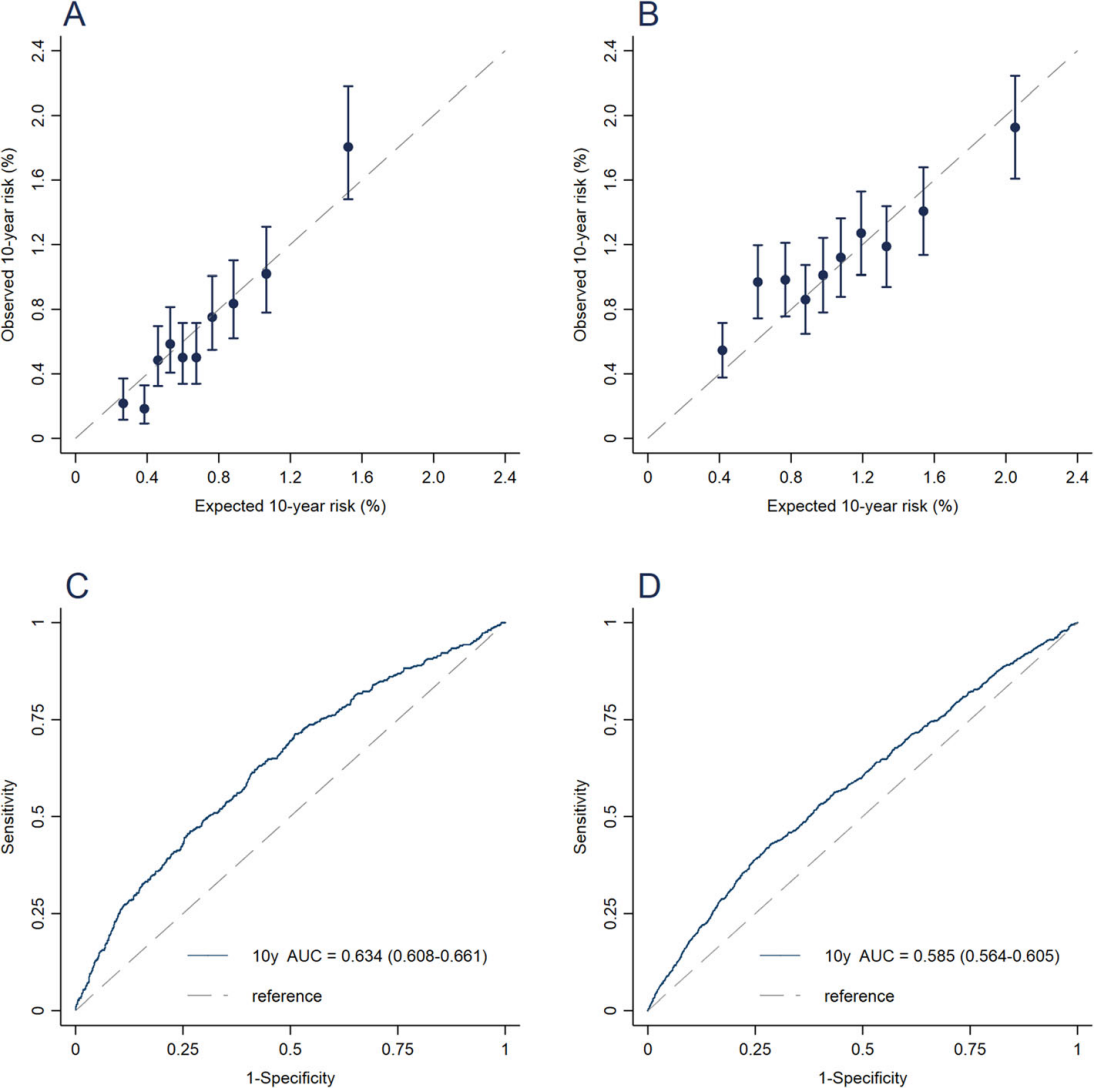
Area under the receiver-operating characteristic curve (AUC) and calibration plot for 10-year breast cancer risk model. Test subcohort of China Kadoorie Biobank (**a**, **c**). Shanghai Women’s Health study (**b**, **d**)

Of the 73,203 women in the SWHS, 1409 were diagnosed with breast cancer during a median of 16.1 years of follow-up. The CKB model predicted 1320 cases in the SWHS, yielding an *E*/*O* of 0.94 (95% CI, 0.89 to 0.99). The number of cases was statistically significantly underestimated among women aged 60 years and older, women with lower education, women shorter than 150.2 cm, women without family history of overall cancer, women with multiple live births, and women with age at menarche at 15–16 years. The model statistically significantly overestimated risk for women with 2 or more affected first-degree relatives. For all other categories, there was good agreement between the observed and predicted number of breast cancers (Table 3). The calibration plot showed agreement across deciles of predicted risk, except for the second-lowest decile (Fig. 1b). We further recalculated the absolute risk using Shanghai local rates and found a better calibration, with an *E*/*O* (95% CI) overall of 1.01 (0.96–1.06) (see Additional file 4).

**Table 3:** Expected and observed number of breast cancer in test subcohort of China Kadoorie Biobank (CKB) and Shanghai Women’s Health Study (SWHS)

	Test subcohort of CKB			SWHS		
	*E*	*O*	*E*/*O* (95% CI)	*E*	*O*	*E*/*O* (95% CI)
Overall	760	751	1.01 (0.94–1.09)	1320	1409	0.94 (0.89–0.99)
Age at enrollment, years						
< 50	372	357	1.04 (0.94–1.16)	735	688	1.07 (0.99–1.15)
50–59	277	256	1.08 (0.96–1.23)	377	394	0.96 (0.87–1.06)
≥ 60	111	138	0.81 (0.68–0.96)	208	327	0.64 (0.57–0.71)
Residence						
Rural	351	267	1.31 (1.17–1.49)	--	--	--
Urban	409	484	0.85 (0.77–0.93)	1320	1409	0.94 (0.89–0.99)
Highest education						
Primary school or lower	333	299	1.11 (0.99–1.25)	143	195	0.73 (0.64–0.85)
Middle school	218	212	1.03 (0.90–1.18)	487	503	0.97 (0.89–1.06)
High school or higher	209	240	0.87 (0.77–0.99)	690	711	0.97 (0.90–1.05)
BMI, kg/m2						
< 18.5	21	17	1.21 (0.76–2.08)	34	38	0.90 (0.66–1.27)
18.5–23.9	340	326	1.04 (0.94–1.17)	627	660	0.95 (0.88–1.03)
24.0–27.9	282	282	1.00 (0.89–1.13)	477	512	0.93 (0.85–1.02)
≥ 28	117	126	0.93 (0.78–1.12)	181	199	0.91 (0.79–1.05)
Height, cm						
< 150.2	139	122	1.14 (0.96–1.38)	85	119	0.71 (0.59–0.86)
150.2–154.1	172	151	1.14 (0.97–1.35)	185	199	0.93 (0.81–1.07)
154.2–158.1	204	217	0.94 (0.82–1.08)	360	387	0.93 (0.84–1.03)
≥ 158.2	245	261	0.94 (0.83–1.06)	690	704	0.98 (0.91–1.06)
No. of first-degree relatives diagnosed with overall cancer						
0	607	573	1.06 (0.98–1.15)	926	1023	0.91 (0.85–0.96)
1	126	144	0.87 (0.74–1.04)	316	329	0.96 (0.86–1.07)
≥ 2	27	34	0.80 (0.57–1.16)	77	57	1.35 (1.04–1.78)
No. of live birth						
Nulliparous	14	12	1.17 (0.67–2.26)	52	58	0.89 (0.69–1.18)
1	340	353	0.96 (0.87–1.07)	852	822	1.04 (0.97–1.11)
≥2	406	386	1.05 (0.95–1.17)	416	529	0.79 (0.72–0.86)
Age at menarche, years						
< 12	54	60	0.90 (0.70–1.19)	118	105	1.12 (0.93–1.37)
13–14	244	266	0.92 (0.81–1.04)	556	567	0.98 (0.90–1.07)
15–16	279	275	1.01 (0.90–1.15)	481	553	0.87 (0.80–0.95)
≥ 17	183	150	1.22 (1.04–1.45)	164	184	0.89 (0.77–1.04)

Abbreviations: *BMI* body mass index, *PY* person-year, *RR* relative risk, *CI* confidence interval, *E* expected number of cases, *O* observed number of cases, -- not applicable

As a reference, we also present calibration results for the test subcohort of the CKB study (Table 3 and Fig. 1a). Overall, the CKB model predicted 760 cases in the CKB test subcohort, yielding an *E*/*O* (95% CI) of 1.01 (0.94–1.09). The model statistically significantly overestimated the risk of women in rural areas but underestimated the risk in urban areas. In the sensitivity analysis, we recalculated the absolute risk using CKB rates (see Additional file 4), and found the calibrated E/Os were 1.03 (0.95–1.13) and 0.99 (0.88–1.12) for participants in the urban and rural areas, respectively.

Discriminating accuracy of the 10-year risk model is presented in Table 4 and Fig. 1c, d. The overall AUC was 0.658 (95% CI, 0.631–0.684) in the CKB test subcohort and attenuated to 0.634 (95% CI, 0.608–0.661) after adjusting for age and residence. External validation resulted in an overall unadjusted AUC of 0.573 (95% CI, 0.553–0.593) and an age-adjusted AUC of 0.585 (95% CI, 0.564–0.605).

**Table 4:** Discrimination of the CKB 10-year prediction model in the test subcohort of China Kadoorie Biobank (CKB) and Shanghai Women’s Health Study (SWHS)

	Test subcohort of CKB	SWHS
	AUCs (95% CIs)	AUCs (95% CIs)
Basic model^a^		
Overall	0.609 (0.581 to 0.637)	0.505 (0.487 to 0.524)
Residence-adjusted	0.533 (0.506 to 0.561)	--
Full model		
Overall^b^	0.658 (0.631 to 0.684)	0.573 (0.553 to 0.593)
Age-specific, year		
< 50		
30–34	0.542 (0.346 to 0.738)	--
35–39	0.602 (0.520 to 0.684)	--
40–44	0.661 (0.600 to 0.723)	0.562 (0.522 to 0.601)
45–49	0.579 (0.502 to 0.657)	0.545 (0.500 to 0.589)
≥ 50		
50–54	0.638 (0.578 to 0.698)	0.566 (0.511 to 0.621)
55–59	0.594 (0.515 to 0.673)	0.611 (0.558 to 0.665)
60–64	0.682 (0.595 to 0.770)	0.604 (0.545 to 0.662)
65–69	0.740 (0.636 to 0.844)	0.673 (0.622 to 0.723)
70–74	0.777 (0.693 to 0.862)	--
75–79	0.960 (0.927 to 0.994)	--
Residence-specific		
Urban	0.646 (0.614 to 0.679)	--
Rural	0.615 (0.568 to 0.661)	--
Age- and residence-adjusted^c^	0.634 (0.608 to 0.661)	0.585 (0.564 to 0.605)

Abbreviations: *AUC* area under the receiver characteristic operating curve, *CI* confidence interval, -- not applicable

^a^Basic model included age and residence in the CKB and included age only in the SWHS

^b^Overall AUC indicated the discriminating ability of the absolute risk predicted by our full model

^c^Age- and residence-adjusted AUC was estimated by testing the full model while adjusting for residence (urban/rural) and age at entry in a 5-year interval, i.e., the prediction effect of age and residence was removed

And compared with women in the lowest quintile of 10-year predicted risk, the adjusted RR for women in the highest quintile was 6.74 in the CKB (95% CI, 4.57–9.92) and 2.55 in the SWHS (95% CI, 2.06–3.16) (Table 5). Larger RRs were observed in women aged 50 years and older and women in urban areas. The stratifying efficiency of our model at different 10-year predicted risk cut-offs in the CKB and SWHS is shown in Additional files 5 and 6.

**Table 5:** Age- and residence-adjusted RR (95% CI) by quantiles of predicted risk in the test subcohort of China Kadoorie Biobank (CKB) and Shanghai Women’s Health Study (SWHS)

	Overall		Urban		Rural		Age < 50 years		Age ≥ 50 years	
	Cases	RR (95% CI)	Cases	RR (95% CI)	Cases	RR (95% CI)	Cases	RR (95% CI)	Cases	RR (95% CI)
Test subcohort of CKB (*n* = 100,274)										
1^a^	46	1.00 (reference)	16	1.00 (reference)	30	1.00 (reference)	18	1.00 (reference)	28	1.00 (reference)
2	96	2.23 (1.53 to 3.24)	38	2.07 (1.15 to 3.76)	58	2.03 (1.21 to 3.39)	46	1.32 (0.76 to 2.32)	50	2.76 (1.64 to 4.64)
3	105	2.60 (1.77 to 3.82)	52	2.44 (1.34 to 4.44)	53	2.11 (1.22 to 3.65)	52	1.30 (0.72 to 2.32)	53	3.38 (1.97 to 5.82)
4	172	4.15 (2.84 to 6.08)	104	3.79 (2.11 to 6.83)	68	3.26 (1.87 to 5.71)	94	2.24 (1.25 to 4.01)	78	4.61 (2.67 to 7.95)
5	277	6.74 (4.57 to 9.92)	237	5.72 (3.16 to 10.33)	40	5.21 (2.85 to 9.50)	121	2.87 (1.55 to 5.30)	156	8.30 (4.81 to 14.31)
SWHS (*n* = 73,203)										
1	185	1.00 (reference)	--	--	--	--	29	1.00 (reference)	156	1.00 (reference)
2	237	1.36 (1.10 to 1.68)	--	--	--	--	145	1.00 (0.67 to 1.49)	92	1.38 (1.06 to 1.81)
3	274	1.62 (1.31 to 2.02)	--	--	--	--	181	1.11 (0.75 to 1.65)	93	1.87 (1.42 to 2.46)
4	308	1.89 (1.53 to 2.35)	--	--	--	--	182	1.32 (0.89 to 1.97)	126	2.07 (1.58 to 2.70)
5	405	2.55 (2.06 to 3.16)	--	--	--	--	151	1.61 (1.06 to 2.43)	254	3.00 (2.34 to 3.84)

Abbreviations: *RR* relative risk, *CI* confidence interval, -- not applicable

Cox model was stratified by age in a 5-year interval in SWHS and additionally stratified by 10 study sites in CKB

^a^1 refers to the lowest risk group, and 5 refers to the highest risk group

## Discussion

We developed a prediction model for invasive breast cancer among Chinese women aged 30 years and older using data from a large nationwide prospective cohort and validated its performance in an independent cohort in Shanghai. The model includes six factors in the relative risk prediction (education, BMI, height, family history of overall cancer, parity, and age at menarche) and two additional factors in the absolute risk prediction (age and residence area). The model was well-calibrated in both the CKB and SWHS cohorts, though there were under- or overestimation of risk in some risk factor strata. After eliminating the effect of age and residence, we found the adjusted AUC was 0.634 and 0.585 in the CKB and SWHS, respectively, which are comparable with those of some previous externally validated models [9, 25].

Overall, our model fits well in the CKB and underestimated (6%) the risk of women in the urban area in the SWHS. To have a good model generalization, we have applied China’s national age and residence (urban/rural) rates in the absolute risk calculation, instead of regional rates like previous studies in China [9–15]. Therefore, the agreement of the national rates with rates in validation datasets may play a major role in the calibration. CKB’s cancer incidence and mortality rates were consistent with national rates during 2008–2013 [26], resulting in the excellent calibration in the CKB. Despite the overall concordance, the model overestimated the risk of women in rural areas but underestimated the risk in urban areas, reflecting that higher incidence rates in urban areas and lower rates in rural areas in the CKB cohort than the corresponding national rates (see Additional file 2). Interestingly, although SWHS cohort women were recruited around 10 years before the CKB in Shanghai, one of the most developed cities in China, the CKB model can still provide acceptable calibration in the SWHS cohort. The slight underestimation was caused by higher incidence rates of breast cancer in Shanghai. In our sensitivity analyses of recalculating the absolute risk using local rates, the above-mentioned calibration errors diminished, confirming that our relative risk model was robust and the errors were solely caused by the mismatch between national rates and local rates (see Additional file 4). A previous meta-analysis showed that the Asian American Breast Cancer Study model (AABCS), or Gail model for Asian Americans, overestimated breast cancer risk for Asian women (pooled *E*/*O* = 1.82, 95% CI 1.31–2.51) [7, 8]. This overestimation was also observed in a recent cohort study in China (*E*/*O* = 2.39, 95% CI 1.71–3.46) [9]. Similarly, we applied the AABCS model to the CKB and SWHS data and found an E/O of 1.89 (95% CI, 1.82–1.97) and 1.16 (1.10–1.23) for the CKB and SWHS, respectively. We further recalibrated the AABSC model using rates from China and still found an overall miscalibration (CKB: *E*/*O* [95% CI], 0.94 [0.90–0.98]; SWHS: 0.67 [0.63–0.71]) and for most subgroups defined by the predicted risk deciles (see Additional file 7).

In the external validation, we found a moderate AUC of 0.585, which was better than or equivalent to those of the AABCS model [8, 9, 25]. Matsuno et al. reported the AUC of the AABCS model (including age at menarche, age at first live birth, number of affected mothers, sisters, and daughters with breast cancer, and number of previous benign biopsies) was 0.614 (95% CI 0.587–0.640) in the validation among Asian-Americans [7], but AUC decreased to 0.54 in two independent validations conducted in China [9] and Korean [25]. We found that the age- and residence-adjusted AUCs of both the original AABCS model and calibrated AABCS model in the CKB and the SWHS data were all around 0.54 (see Additional file 7). To our knowledge, only one model developed in China was externally validated, with higher AUC (0.64, 95% CI 0.55–0.72), but few cases in their validation set and same location of derivation and validation sets limited the robustness of the results [9]. Although several models in China had statistically significantly higher AUC by additionally including genetic information, the lack of external validation precludes direct comparison with our models [11, 14, 15].

The development of the CKB risk prediction model has several public health implications. First, our model, with the moderate discriminating ability and good calibration, can facilitate allocation of preventive resources under monetary and medical constraints and aid risk-based screening strategies [27]. China’s breast cancer 2019 screening guidelines recommended an opportunity for screening for women with average risk aged 40–44 years and biennial screening for women aged 45–69 years, which is mainly done by mammograph and supplemented with breast ultrasonography and magnetic resonance imaging [28]. However, such an age-based screening strategy ignores the large variation in breast cancer risk in the population [29]. Given the limited medical and economic resources in China, it is more cost-effective to adopt a risk-based screening strategy that can allocate resources to do intensive screening for women at high risk, while less frequent screening for women with low risk. Second, at the individual level, our model can be used for individual risk counseling and promote a healthy lifestyle. Knowing their own cancer risk may motivate obese women to lose weight. Third, as described by Gail et al., our model can also aid designing preventive trials and estimating the absolute burden of a specific population [27].

Our study has several strengths. We used data from the largest nationwide prospective cohort study in China to develop the relative risk model, augmented with China national incidence and mortality rates, and validated in another large prospective cohort study. These methods ensure our model to be robust and potentially generalizable to both rural and urban areas in China. Also, all predictors in the model are non-invasive, easy to measure at low cost, which makes the model easily applicable to the general population. We plan to develop an online risk calculator to promote its use.

However, one must be aware of limitations of our study. First, several established risk factors were not included in the model. Although several studies included alcohol [29–31], the low prevalence of alcohol intake in the CKB (see Table 1) precluded the inclusion. Additionally, we did not have data on family history of breast cancer, so we used a family history of all cancers as a surrogate to capture the inherited susceptibility of breast cancer as much as possible. This surrogation may not be accurate such that the risk was overestimated in women with two or more family members having cancers*.* The history of benign breast diseases was not collected in the CKB and we think it might not be reliably collected in the general Chinese population. Second, cumulative evidence showed heterogeneous associations of epidemiological factors with estrogen receptor (ER)-specific breast cancer though some factors are common for both ER-positive and ER-negative breast cancers [32, 33]. We did not build ER-specific models due to the lack of information on subtypes of breast cancer in the current database of the CKB cohort. Since the majority of breast cancer in Chinese women was estrogen ER-positive (80.3% in women < 50 years and 76.8% in women 50 or older) [34], our model might primarily apply to ER-positive breast cancer. Finally, we only externally validated our model in urban Shanghai, which has one of the highest incidence rates in China. Therefore, further validation of our model in other regions, especially in rural regions, is still needed.

## Conclusions

In summary, we have developed and validated a breast cancer risk prediction model that only relies on non-laboratory predictors. The model has a good calibration and a moderate discriminating capacity. The model may serve as a useful tool to raise individuals’ awareness and to identify women who may benefit from breast cancer screening in China. To improve the model discriminating accuracy, further studies can add genetic and epigenetic predictors for breast cancer, as well as mammographic density. Validation of our model in other regions of China, especially rural areas, is also desirable to evaluate the robustness of the CKB model.

## Supplementary Information

**Additional file 1.** Show comparison of Age-adjusted RR (95% CI) for breast cancer among women in urban and rural areas of China Kadoorie Biobank.

**Additional file 2.** Show age- and residence-specific breast cancer incidence rates and mortality rates of non-breast cancer per 100,000 person-years by data sources.

**Additional file 3.** Show age- and site-adjusted RR (95% CI) from the derivation subcohort and the whole China Kadoorie Biobank.

**Additional file 4.** Show expected and observed number of breast cancer in the test subcohort of China Kadoorie Biobank and Shanghai Women’s Health Study using the corresponding local rates.

**Additional file 5.** Show performance of the breast cancer prediction model across different predicted risk cutoffs in the China Kadoorie Biobank.

**Additional file 6.** Show performance of the breast cancer prediction model across different predicted risk cutoffs in the Shanghai Women's Health Study.

**Additional file 7.** Show validation of the Asian America Breast Cancer Study model for predicting individual breast cancer risk in China Kadoorie Biobank and Shanghai Women's Health Study.

## Data Availability

Details of how to access China Kadoorie Biobank data and details of the data release schedule are available from www.ckbiobank.org/site/Data+Access.

## Abbreviations

AABCS: Asian American Breast Cancer Study model
AUC: Area under the receiver-operating characteristic curve
BIC: Bayesian Information Criterion
BMI: Body mass index
CI: Confidence interval
CKB: China Kadoorie Biobank
E: Expected number of breast cancer cases
ICD-10: International Classification of Diseases, 10th Revision
NCCR: National Central Cancer Registry
NNS: Numbers needed to be screened to confirm one case
NPV: Negative predictive value
O: Observed number of breast cancer cases
PPV: Positive predictive value
RR: Relative risk
SWHS: Shanghai Women’s Health Study
PAR: Population attributable risk
