# Supplementary material for: Development and external validation of a breast cancer absolute risk prediction model in Chinese population
Source: Breast Cancer Res. 2021 May 29;23:62. doi: 10.1186/s13058-021-01439-2 (PMC8164768; doi:10.1186/s13058-021-01439-2)
Supplement: Supplementary file 1 — Additional file 1. Show comparison of Age-adjusted RR (95% CI) for breast cancer among women in urban and rural areas of China Kadoorie Biobank. [file 13058_2021_1439_MOESM1_ESM.pdf]

Additional file 1. Comparison of Age-adjusted RR (95% CI) for breast cancer among women in urban and rural areas of China Kadoorie Biobank

	Urban			Rural		
	Cases	Cases/PYs (/10,000)	RR (95% CI)	Cases	Cases/PYs (/10,000)	RR (95% CI)
Highest education						
No formal school	126	54.98	1.00 (reference)	213	40.17	1.00 (reference)
Primary school	257	96.41	1.17 (1.10 to 1.24)	313	45.99	1.17 (1.07 to 1.29)
Middle school	454	113.97	1.36 (1.21 to 1.53)	199	53.57	1.37 (1.14 to 1.65)
High school	448	145.2	1.59 (1.34 to 1.89)	57	58.71	1.61 (1.22 to 2.12)
College/university	206	168.25	1.85 (1.47 to 2.33)	14	130.35	1.89 (1.31 to 2.73)
BMI at attained age < 50 years, kg/m^2						
<18.5	10	111.76	1.00 (reference)	11	89.43	1.00 (reference)
<24.0	217	198.34	0.92 (0.79 to 1.07)	140	80.93	0.99 (0.83 to 1.19)
<28.0	109	257.99	0.84 (0.62 to 1.14)	78	104.95	0.99 (0.69 to 1.42)
≥28.0	20	169.83	0.77 (0.48 to 1.22)	23	115.65	0.98 (0.57 to 1.69)
BMI at attained age ≥ 50 years, kg/m^2						
<18.5	18	57.96	1.00 (reference)	18	29.43	1.00 (reference)
<24.0	405	101.48	1.25 (1.16 to 1.36)	237	42.02	1.25 (1.12 to 1.40)
<28.0	470	127.47	1.57 (1.34 to 1.84)	213	60.28	1.57 (1.25 to 1.96)
≥28.0	242	158.83	1.97 (1.56 to 2.49)	76	65.23	1.96 (1.40 to 2.74)
Height, cm						
100-	212	84.17	1.00 (reference)	170	34.79	1.00 (reference)
150.2-	299	95.9	1.14 (1.08 to 1.20)	205	46.14	1.13 (1.05 to 1.21)
154.2-	379	109.28	1.29 (1.16 to 1.43)	217	52.67	1.27 (1.10 to 1.46)
158.2-	601	144.97	1.47 (1.25 to 1.72)	204	59.11	1.43 (1.16 to 1.76)
Family history of cancer						
no family history	1,161	108.93	1.00 (reference)	634	44.03	1.00 (reference)
One relative history	270	120.62	1.04 (0.91 to 1.19)	132	60.19	1.24 (1.02 to 1.50)
More than one	60	169.7	1.42 (1.09 to 1.85)	30	97.48	1.96 (1.35 to 2.85)
Live birth count						
Nulliparous	36	141.05	1.86 (1.27 to 2.71)	9	62.61	1.74 (0.89 to 3.41)
1	856	125.38	1.77 (1.42 to 2.21)	211	57.8	1.49 (1.13 to 1.95)
2	366	117.81	1.43 (1.18 to 1.74)	353	53.61	1.38 (1.12 to 1.68)
≥3	233	76.11	1.00 (reference)	223	34.19	1.00 (reference)
Age at menarche, years						
<12	134	160.4	1.50 (1.24 to 1.80)	53	66.67	1.56 (1.21 to 2.00)
13-14	507	126.34	1.31 (1.16 to 1.48)	241	53.85	1.34 (1.14 to 1.59)
15-16	537	112.23	1.14 (1.08 to 1.22)	300	46.27	1.16 (1.07 to 1.26)
≥17	313	86.53	1.00 (reference)	202	39.25	1.00 (reference)

Abbreviations: BMI, body mass index; PY, person-year; RR, relative risk; CI, confidence interval.

Cox model was stratified by 5 study sites and age at baseline in a 5-year interval, and included all the predictors in the table.
