# Supplementary material for: Development and external validation of a breast cancer absolute risk prediction model in Chinese population
Source: Breast Cancer Res. 2021 May 29;23:62. doi: 10.1186/s13058-021-01439-2 (PMC8164768; doi:10.1186/s13058-021-01439-2)
Supplement: Supplementary file 2 — Additional file 2. Show age- and residence-specific breast cancer incidence rates and mortality rates of non-breast cancer per 100,000 person-years by data sources. [file 13058_2021_1439_MOESM2_ESM.pdf]

Additional file 2. Age- and residence-specific breast cancer incidence rates and mortality rates of non-breast cancer per 100,000 person-years by data sources

	CKB				Nation				Shanghai	
	Urban		Rural		Urban		Rural		Urban	
	h_1	h_2	h_1	h_2	h_1	h_2	h_1	h_2	h_1	h_2
30-34	98.4	0.0	0.0	63.9	16.7	35.5	13.2	52.2	10.2	44.3
35-39	48.5	31.6	22.4	116.8	34.2	43.2	27.1	62.1	31.1	65.3
40-44	82.0	71.5	38.1	101.7	64.8	80.2	54.1	96.8	69.7	83.1
45-49	113.9	100.0	52.1	170.5	85.7	101.1	70.8	134.7	105	128.1
50-54	122.1	160.6	57.2	250.8	105.2	225.6	71.5	268.5	115.2	203.1
55-59	123.2	222.9	53.7	427.5	107.7	275.9	73.6	344.3	109.9	317.5
60-64	138.6	382.0	52.3	690.5	108.6	606.4	73.2	683.5	106.6	530.9
65-69	118.7	687.1	40.4	1208.0	98.7	1105.4	52.3	1285.2	103.3	993.7
70-74	100.6	1274.6	38.0	2152.1	92.5	1843.2	46.0	2225.7	113.3	1838.1
75-79	100.5	2422.5	34.6	3879.7	83.4	3482.2	39.0	3821.9	106	3547.3
80-84	81.7	4346.7	21.7	6699.8	74.9	6984.1	30.0	6776.3	74.9	6984.1
85-89	103.7	7925.7	157.9	7890.3	47.8	16282.3	22.1	14950.6	47.8	16282.3

Abbreviation: CKB, China Kadoorie Biobank; h_1, incidence rate of breast cancer; h_2, mortality rates of non-breast cancer.

CKB's rates were estimated among participants included in model derivation (2004–2016, n=300,824). National rates were calculated using incidence rates and breast cancer-specific mortality rates in 2014 from National Central Cancer Registry of China and all-cause mortality rates in 2014 from Health Statistics Yearbook. Shanghai's rates in age groups 30-79 were obtained from Zheng W, Wen W, Gao YT, et al. J Natl Cancer Inst 2010;102:972-81, while the rates in age groups 80-89 were replaced with national rates as they are not available in Zheng W et al.
