# Supplementary material for: Development and external validation of a breast cancer absolute risk prediction model in Chinese population
Source: Breast Cancer Res. 2021 May 29;23:62. doi: 10.1186/s13058-021-01439-2 (PMC8164768; doi:10.1186/s13058-021-01439-2)
Supplement: Supplementary file 3 — Additional file 3. Show age- and site-adjusted RR (95% CI) from the derivation subcohort and the whole China Kadoorie Biobank. [file 13058_2021_1439_MOESM3_ESM.pdf]

Additional file 3. Age- and site-adjusted RR (95% CI) from the derivation subcohort and the whole China Kadoorie Biobank

	Derivation subcohort			Whole cohort		
	Cases	Cases/PYs (/100,000)	RR (95% CI)	Cases	Cases/PYs (/100,000)	RR (95% CI)
Highest education						
No formal school	222	43.65	1.00 (Reference)	339	44.64	1.00 (Reference)
Primary school	388	61.47	1.19 (1.12 to 1.26)	570	60.18	1.17 (1.11 to 1.23)
Middle school	441	86.19	1.41 (1.25 to 1.58)	653	84.82	1.37 (1.24 to 1.51)
High school	339	125.95	1.67 (1.40 to 1.99)	505	124.5	1.60 (1.39 to 1.85)
College/university	146	163.88	1.98 (1.56 to 2.50)	220	165.2	1.87 (1.54 to 2.27)
BMI at age < 50 years, kg/m^2						
<18.5	17	118.09	1.00 (Reference)	21	98.83	1.00 (Reference)
18.5-23.9	235	124.68	0.97 (0.84 to 1.12)	357	126.42	0.95 (0.84 to 1.07)
24.0-27.9	116	148.92	0.95 (0.71 to 1.26)	187	160.42	0.90 (0.71 to 1.14)
≥28	35	164.52	0.92 (0.60 to 1.41)	43	135.81	0.85 (0.60 to 1.21)
BMI at age ≥ 50 years, kg/m^2						
<18.5	23	39.28	1.00 (Reference)	36	41.07	1.00 (Reference)
18.5-23.9	438	68.05	1.23 (1.14 to 1.33)	642	66.66	1.25 (1.17 to 1.33)
24.0-27.9	472	98.43	1.51 (1.29 to 1.77)	683	94.59	1.57 (1.38 to 1.78)
≥28	200	111.94	1.86 (1.47 to 2.35)	318	118.27	1.96 (1.62 to 2.38)
Height, cm						
<150.2	260	52.68	1.00 (Reference)	382	51.58	1.00 (Reference)
150.2-154.1	353	69.95	1.12 (1.06 to 1.18)	504	66.66	1.13 (1.09 to 1.18)
154.2-158.1	379	74.94	1.25 (1.13 to 1.38)	596	78.54	1.28 (1.18 to 1.39)
≥158.2	544	107.56	1.40 (1.20 to 1.63)	805	105.96	1.45 (1.28 to 1.65)
No. of first-degree relatives diagnosed with overall cancer						
0	1,222	73.2	1.00 (Reference)	1,795	71.63	1.00 (Reference)
1	258	87.03	1.05 (0.91 to 1.20)	402	90.71	1.10 (0.99 to 1.23)
≥2	56	128.2	1.46 (1.11 to 1.91)	90	136.09	1.57 (1.27 to 1.95)
No. of live birth						
Nulliparous	33	124.9	1.84 (1.26 to 2.68)	45	112.79	1.78 (1.29 to 2.45)
1	714	102.53	1.57 (1.28 to 1.93)	1,067	101.84	1.66 (1.40 to 1.96)
2	476	73.41	1.32 (1.12 to 1.57)	719	74.19	1.41 (1.22 to 1.62)
≥3	313	49.03	1.00 (Reference)	456	47.58	1.00 (Reference)
Age at menarche, years						
<12	127	117.02	1.39 (1.16 to 1.67)	187	114.7	1.52 (1.31 to 1.76)
13-14	482	85.18	1.25 (1.10 to 1.41)	748	88.12	1.32 (1.20 to 1.46)
15-16	562	74.85	1.12 (1.05 to 1.19)	837	74.27	1.15 (1.09 to 1.21)
≥17	365	62.46	1.00 (Reference)	515	58.77	1.00 (Reference)

Abbreviations: BMI, body mass index; PY, person-year; RR, relative risk; CI, confidence interval.

Cox model was stratified by study sites (10 areas) and age at baseline in 5-year interval, and included all the predictors in the table.
