# Supplementary material for: Development and external validation of a breast cancer absolute risk prediction model in Chinese population
Source: Breast Cancer Res. 2021 May 29;23:62. doi: 10.1186/s13058-021-01439-2 (PMC8164768; doi:10.1186/s13058-021-01439-2)
Supplement: Supplementary file 4 — Additional file 4. Show expected and observed number of breast cancer in the test subcohort of China Kadoorie Biobank and Shanghai Women’s Health Study using the corresponding local rates. [file 13058_2021_1439_MOESM4_ESM.pdf]

Additional file 4. Expected and observed number of breast cancer in the test subcohort of China Kadoorie Biobank (CKB) and Shanghai Women's Health Study (SWHS) using the corresponding local rates

	Test subcohort of CKB[a]			SWHS[b]		
	E	*O*	*E/O* (95% CI)	*E*	*O*	*E/O* (95% CI)
Overall	765	751	1.02 (0.95-1.10)	1,423	1,409	1.01 (0.96-1.06)
Age at enrollment, years						
<50	372	357	1.04 (0.94-1.16)	793	688	1.15 (1.07-1.24)
50-59	278	256	1.08 (0.96-1.23)	391	394	0.99 (0.90-1.10)
≥60	115	138	0.84 (0.71-0.99)	239	327	0.73 (0.65-0.82)
Residence						
Rural	264	267	0.99 (0.88-1.12)	--	--	--
Urban	501	484	1.03 (0.95-1.13)	--	--	--
Highest education						
Primary school or lower	299	299	1.00 (0.89-1.12)	160	195	0.82 (0.71-0.95)
Middle school	226	212	1.07 (0.93-1.23)	522	503	1.04 (0.95-1.14)
High school or higher	239	240	1.00 (0.88-1.14)	740	711	1.04 (0.97-1.12)
BMI, kg/m^2						
<18.5	19	17	1.13 (0.71-1.94)	37	38	0.98 (0.72-1.39)
18.5-23.9	334	326	1.03 (0.92-1.15)	678	660	1.03 (0.95-1.11)
24.0-27.9	288	282	1.02 (0.91-1.15)	513	512	1.00 (0.92-1.10)
≥28	123	126	0.98 (0.82-1.18)	195	199	0.98 (0.85-1.13)
Height, cm						
<150.2	127	122	1.04 (0.87-1.26)	93	119	0.78 (0.65-0.94)
150.2-154.1	168	151	1.11 (0.95-1.31)	200	199	1.01 (0.88-1.16)
154.2-158.1	206	217	0.95 (0.83-1.09)	389	387	1.00 (0.91-1.11)
≥158.2	263	261	1.01 (0.89-1.14)	741	704	1.05 (0.98-1.13)
No. of first-degree relatives diagnosed with overall cancer						
0	598	573	1.04 (0.96-1.14)	1,000	1,023	0.98 (0.92-1.04)
1	136	144	0.95 (0.80-1.12)	340	329	1.03 (0.93-1.15)
≥2	30	34	0.89 (0.64-1.28)	83	57	1.45 (1.12-1.91)
No. of live birth						
Nulliparous	14	12	1.18 (0.68-2.29)	56	58	0.96 (0.75-1.27)
1	376	353	1.07 (0.96-1.19)	914	822	1.11 (1.04-1.19)
≥2	374	386	0.97 (0.88-1.07)	453	529	0.86 (0.79-0.93)
Age at menarche, years						
<12	60	60	1.00 (0.77-1.31)	127	105	1.21 (1.00-1.48)
13-14	255	266	0.96 (0.85-1.09)	599	567	1.06 (0.97-1.15)
15-16	276	275	1.00 (0.89-1.13)	519	553	0.94 (0.86-1.02)
≥17	173	150	1.16 (0.99-1.37)	178	184	0.97 (0.84-1.12)

Abbreviations: BMI, body mass index; PY, person-year; RR, relative risk; CI, confidence interval; E, expected number of cases; O, observed number of cases; --, not applicable.

[a]Absolute risk was recalibrated using the breast cancer incidence rates and non-breast cancer mortality rates in the CKB.

[b]Absolute risk was recalibrated using the breast cancer incidence rates and non-breast cancer mortality rates in Shanghai (Additional file 2).
