# Supplementary material for: Development and external validation of a breast cancer absolute risk prediction model in Chinese population
Source: Breast Cancer Res. 2021 May 29;23:62. doi: 10.1186/s13058-021-01439-2 (PMC8164768; doi:10.1186/s13058-021-01439-2)
Supplement: Supplementary file 6 — Additional file 6. Show performance of the breast cancer prediction model across different predicted risk cutoffs in the Shanghai Women's Health Study. [file 13058_2021_1439_MOESM6_ESM.pdf]

Additional file 6. Performance of the breast cancer prediction model across different predicted risk cutoffs in the Shanghai Women's Health Study

Cutoff of 10-year risk (%)	Percent of high-risk population (%)[a]	Sensitivity (%)	Specificity (%)	Positive predictive value (%)	Negative predictive value (%)	Number needed to be screened to identify one case
0.4	96.57	98.91	3.46	1.21	99.63	82.86
0.5	92.15	96.00	7.90	1.23	99.40	81.46
0.6	87.08	91.77	12.98	1.24	99.25	80.53
0.7	81.44	86.68	18.62	1.25	99.15	79.74
0.8	73.96	80.15	26.12	1.28	99.10	78.32
0.9	64.42	72.28	35.68	1.32	99.08	75.64
1.0	54.00	63.44	46.11	1.38	99.06	72.25
1.1	43.75	54.00	56.38	1.45	99.04	68.76
1.2	34.96	44.79	65.16	1.51	99.00	66.23
1.3	27.55	37.17	72.57	1.59	98.98	62.90
1.4	21.53	30.51	78.58	1.67	98.96	59.88
1.5	16.74	25.06	83.36	1.76	98.94	56.70
1.6	12.95	20.58	87.14	1.87	98.92	53.41
1.7	9.89	16.46	90.19	1.96	98.91	50.97
1.8	7.47	13.08	92.59	2.06	98.89	48.51
1.9	5.59	9.56	94.46	2.02	98.87	49.61
2.0	4.37	7.75	95.67	2.09	98.86	47.86

[a]High-risk population is defined as people whose 10-year predicted risk are higher than the corresponding cutoff.
