# Supplementary material for: Development and external validation of a breast cancer absolute risk prediction model in Chinese population
Source: Breast Cancer Res. 2021 May 29;23:62. doi: 10.1186/s13058-021-01439-2 (PMC8164768; doi:10.1186/s13058-021-01439-2)
Supplement: Supplementary file 7 — Additional file 7. Show validation of the Asian America Breast Cancer Study model for predicting individual breast cancer risk in China Kadoorie Biobank and Shanghai Women's Health Study. [file 13058_2021_1439_MOESM7_ESM.pdf]

Additional file 7. Validation of the Asian America Breast Cancer Study model (AABCS) for predicting individual breast cancer risk in China Kadoorie Biobank (CKB) and Shanghai Women's Health Study (SWHS)

	**CKB**					SWHS				
	**Overall AUC[a]**	**Adjusted AUC[b]**	***E***	***O***	***E/O* (95% CI)**	**Overall AUC[a]**	**Adjusted AUC[b]**	**E**	***O***	***E/O* (95% CI)**
Original AABCS model										
Overall	0.574 (0.558-0.590)	0.543 (0.527-0.559)	4,325	2,287	1.89 (1.82-1.97)	0.553 (0.532-0.573)	0.556 (0.535-0.576)	1,639	1,409	1.16 (1.10-1.23)
1	--	--	180	154	1.17 (1.00-1.38)	--	--	102	117	0.87 (0.73-1.05)
2	--	--	250	181	1.38 (1.19-1.60)	--	--	132	125	1.06 (0.89-1.27)
3	--	--	276	162	1.70 (1.46-2.00)	--	--	143	132	1.08 (0.91-1.30)
4	--	--	318	212	1.50 (1.31-1.73)	--	--	140	117	1.19 (1.00-1.44)
5	--	--	354	181	1.96 (1.69-2.27)	--	--	147	121	1.22 (1.02-1.47)
6	--	--	363	193	1.88 (1.63-2.18)	--	--	161	132	1.22 (1.03-1.45)
7	--	--	406	269	1.51 (1.34-1.71)	--	--	176	131	1.35 (1.13-1.61)
8	--	--	465	292	1.59 (1.42-1.79)	--	--	181	163	1.11 (0.95-1.30)
9	--	--	599	306	1.96 (1.75-2.20)	--	--	200	169	1.18 (1.02-1.38)
10	--	--	1115	337	3.31 (2.97-3.69)	--	--	258	202	1.28 (1.11-1.47)
Calibrated AABCS model[c]										
Overall	0.608 (0.0.592-0.623)	0.544 (0.529-0.590)	2,147	2,287	0.94 (0.90-0.98)	0.550 (0.530-0.570)	0.555 (0.535-0.575)	942	1,409	0.67 (0.63-0.71)
1	--	--	81	108	0.75 (0.62-0.91)	--	--	52	116	0.44 (0.37-0.54)
2	--	--	110	148	0.74 (0.63-0.88)	--	--	61	107	0.57 (0.47-0.70)
3	--	--	128	136	0.94 (0.80-1.13)	--	--	78	134	0.58 (0.49-0.69)
4	--	--	142	150	0.95 (0.81-1.12)	--	--	83	110	0.75 (0.63-0.92)
5	--	--	153	202	0.76 (0.66-0.87)	--	--	90	149	0.61 (0.52-0.72)
6	--	--	181	207	0.88 (0.76-1.01)	--	--	97	145	0.67 (0.57-0.79)

7	--	--	206	256	0.80 (0.71-0.91)	--	--	104	145	0.71 (0.61-0.85)
8	--	--	255	336	0.76 (0.68-0.85)	--	--	106	140	0.76 (0.64-0.90)
9	--	--	305	363	0.84 (0.76-0.93)	--	--	120	159	0.75 (0.64-0.88)
10	--	--	588	381	1.54 (1.39-1.71)	--	--	153	204	0.75 (0.65-0.86)

Abbreviations: AUC, area under the curve; E, expected number of cases; O, observed number of cases; CI, confidence interval; --, not applicable.

[a]Overall AUC indicated the discriminating ability of the absolute risk predicted by our model.

[b]Adjusted AUC were estimated adjusting for residence (urban/rural) and 10 age groups at entry, i.e. the prediction effect of age and residence was removed.

[c]Calibrated using the breast cancer incidence rates and non-breast cancer mortality rates in China.
